# Serum selenium and selenoprotein-P levels in autoimmune thyroid diseases patients in a select center: a transversal study

**DOI:** 10.1590/2359-3997000000309

**Published:** 2017-12-01

**Authors:** Marco Aurélio Ferreira Federige, João Hamilton Romaldini, Ana Beatriz Pinotti Pedro Miklos, Marcia Kiyomi Koike, Kioko Takei, Evandro de Souza Portes

**Affiliations:** 1 Hospital do Servidor Público Estadual São Paulo SP Brasil Endocrinología, Hospital do Servidor Público Estadual (IAMSPE), São Paulo, SP Brasil

**Keywords:** Selenium, selenoprotein P, Graves’ disease, Graves’ ophthalmopathy, Hashimoto's thyroiditis

## Abstract

**Objective:**

Selenium (Se) supplementation has been used to help prevent the progression of Graves’ ophthalmopathy (GO) and autoimmune thyroid diseases (AITD) patients. We investigated Se serum and selenoprotein P (SePP) levels in Graves’ disease (GD) with and without GO, Hashimoto's thyroiditis (HT) patients and in 27 control individuals (C).

**Subjects and methods:**

We studied 54 female and 19 male patients: 19 with GD without GO, 21 GD with GO, 14 with HT and 19 with HT+LT4. Se values were measured using graphite furnace atomic absorption spectrophotometry. Serum SePP levels were measured by ELISA.

**Results:**

Median Se levels were similar among all groups; GD patients: 54.2 (46.5-61.1 μg/L), GO: 53.6 (43.5-60.0 μg/L), HT: 51.9 (44.6-58.5 μg/L), HT+LT4 54.4 (44-63.4) and C group patients: 56.0 (52.4-61.5 μg/L); P = 0.48. However, serum SePP was lower in GO patients: 0.30 (0.15-1.05 μg/mL) and in HT patients: 0.35 (0.2-1.17 μg/mL) compared to C group patients: 1.00 (0.564.21 μg/mL) as well as to GD patients: 1.19 (0.62-2.5 μg/mL) and HT+LT4 patients: 0.7 (0,25-1.95); P = 0.002. Linear regression analysis showed a significant relationship between SePP and TPOAb values (r = 0.445, R^2^ = 0.293; P < 0.0001). Multiple regression analysis found no independent variables related to Se or SePP.

**Conclusion:**

A serum Se concentration was lower than in some other countries, but not significantly among AITD patients. The low serum SePP levels in GO and HT patients seems to express inflammatory reactions with a subsequent increase in Se-dependent protein consumption remains unclear.

## INTRODUCTION

Selenium (Se) is fundamental to cell metabolism as it is incorporated by a group of important proteins known as selenoproteins, each of which plays a critical role in thyroid metabolism. Thus it is no surprise that the thyroid gland contains the highest concentration of Se per gram of tissue (1). Se levels have been shown to be lower in patients with autoimmune thyroid diseases (AITD) and particularly in Graves’ Ophthalmopathy (GO). Se has an effect in AITD as it influences antioxidative protection through peroxidase glutathione action (GPx) and selenoproteins P (SePP), N, S and K. Se supports normal thyroid function directly in the formation and regulation of thyroid hormones through iodothyronine deiodinases (DI) and thioredoxin reductases (TRx) (1-3). Se deficiency intake can negatively influence the activity of several Se-responsive enzymes, particularly DI and a SePP (4,5). Low Se serum levels are also associated with an increased risk of thyroid diseases (6). Wide variations in the amount of Se found in different foods and soils can also cause wide variations in Se serum according to the studied population (Table 1). Se is transported in the circulation mainly by SePP, which is produced in the liver, and is considered the best nutritional biomarker for Se (1,5) SePP also has an antioxidant activity (2,7). It can reduce hydroperoxides, protecting plasma proteins and endothelial cells against oxidative damage (5). SePP is found in almost all body tissues, regulating energy metabolism and insulin resistance (8-10). Furthermore, SePP serum can serve as a Se status indicator (11). In Se deficiency situations the thyroid gland appears to maintain high concentrations of Se, suggesting that there is a retention mechanism that allows maintaining normal thyroid function at the detriment of other cells and tissues (1,12). Recent have shown that in Hashimoto thyroiditis (HT) and in Graves’ disease (GD) have been associated with Se deficiency and that this disability can trigger the mechanism and progression of AITD (12). In HT patients, the predominantly cytotoxic effects are mediated by T lymphocytes where autoantibody production leads to the destruction of thyroid epithelial cells (7,13), eventually causing thyroid hypofunction in GD patients. The presence of a thyroid-stimulating antibody (TRAb) with consequent abnormal overreaction of the gland explains the elevated increase in thyroid hormone levels in the serum (14). The excessive production of ROS observed in AITD may be the geneses of the observed increase in selenoproteins consumption (7,12,13). It has been described that Se deficiency impairs GPx activity and induces apoptosis and cell death by increasing H_2_O_2_ (15). In addition, recent studies have shown that Se supplementation in HT patients improved inflammation with decrease in the concentration of the thyroid peroxidase antibody (TPOAb) and the antithyroglobulin antibody (TgAb). In GD a decrease in TRAb levels was observed. Particularly in GO patients an improvement in clinical activity was also was observed (13,14,16,17). Few studies investigated serum Se concentrations in AITD. The present study evaluated Se serum and SePP concentrations in AITD patients and the likely association with thyroid function parameters.

**Table 1:** Worldwide serum selenium concentrations in normal subjects

Country	Author (reference)	Year	Patients number	Se (μg/L)
Brazil	Saiki and cols. (30)	2007	32	92.7 ± 7*
Turkey	Erdal and cols. (21)	2008	49	83.7 ± 17.3*
England	Rayman and cols. (22)	2008	501	91.3 (89-92)***
Austria	Moncayo and cols. (23)	2008	554	90.5 ± 20.8*
Greece	Charalabopoulos and cols. (25)	2009	120	68.7 ± 4.5*
USA	Combs and cols. (20)	2011	261	142 ± 23.5*
Japan	Muzembo and cols. (2)	2013	20	116 (80-180)**
China	Liu and cols. (26)	2013	1205	52.6 (40-67)**
Denmark	Pedersen and cols. (12)	2013	830	96.8 ± 19.7*
Australia	McDonald and cols. (24)	2013	581	85.6 ± 0.5*
Brazil	Cardoso and cols. (27)	2015	15	50 ± 15*
Brazil	Present study	2015	27	56 (52.4-61.5)**

* Mean ± SD;

** Median and interquartile intervals;

*** Geometric mean.

## SUBJECTS AND METHODS

### Patients

This study included 73 AITD patients (54 female and 19 male) from the ambulatory of Endocrinology of the *Hospital do Servidor Público Estadual* (HSPE) - IAMSPE and included HT patients (n = 14), HT + LT4 (n = 19), GD patients without GO (n = 19) and GD with GO patients defined as having proptosis and Clinical Activity Index (CAS) greater than 1 (n = 21). A control group (C), consisted of 27 individuals without any autoimmune disease, diabetes mellitus, thyroid disease, presenting normal liver and renal function. All individuals resided in the same location of Sao Paulo City, Brazil and were euthyroid at the time of the study. The HT patients had goiters and elevated serumTPOAb and/or TgAb. The HT + LT4 patients had elevated serum TPOAb and/or TgAb and were taking levothyroxine. The 19 GD had goiters and elevated serum TPOAb and/or TgAb but 75% had elevated serum TRAb. All were euthyroid treated with methimazole for at least a year. Among the 21 patients with GO, 10 had been treated with radioiodine therapy and using levothyroxine and the remaining 11 were being treated with methimazole. The distribution of CAS was as follows: CAS 1 (n = 3), CAS 2 (n = 11), CAS 3 (n = 6) and CAS 5 (n = 1). The inclusion criteria were: ambulatory patients with well-defined AITD diagnosis, female patients could not be pregnant or less than 12 months post-partum at the time of study. The exclusion criteria were as follows: (a) use recent of multivitamins; (b) smoking; (c) frequent alcohol intake; (d) regular consumption Brazil nuts; (e) ongoing amiodarone, antidepressants or anticonvulsants therapy; and (f) the presence of other endocrine or autoimmune diseases. All the participants responded a clinical and nutritional questionnaire about Se ingestion in the last month (attached) in order to avoid some bias in the Se measurements (see Supplement 1). The study was approved by the Research Ethics Committee of the IAMSPE (number of 533,774) and all subjects signed a consent agreement.

### Methods

For Se determinations, blood samples were collected in trace tubes (Vacuette, Greiner BioOne Brazil tubes) and centrifuged at 1,500 g for 15 minutes. The serum was frozen at −20°C. Measurements were performed by atomic absorption spectrometry (Perkin-Elmer model; Perker-Elemer Corp., Norwalk, CT, USA) with a graphite furnace (18). For the SePP determination, blood samples were collected in tubes without anticoagulants, centrifuged at 1,500 g for 15 minutes and the serum then aliquoted into a cryogenic tube and frozen at −80°C until analyses. SePP serum concentrations were determined by sandwich enzyme immunoassay (19) using an USCN Life Science kit (Wuhan, China). All determinations were performed in duplicate The intra-assay coefficient variation (CV) was 4.7% with the inter-assay CV at 8.2%. Blood samples for determinations of thyroid-stimulating antibody (TSH), thyroid hormones, TPOAb, TgAb and TRAb were collected in tubes without anticoagulants, centrifuged and serum aliquot at −20°C until analyses. The biochemical analyses were performed on the same day. The determinations of free thyroxine (FT4), free triiodothyronine (FT3), and TSH were performed by chemiluminescence assay (Unicel DXI 800 Beckman Coulter Inc., USA). Serum TPOAb and TgAb were determined by chemiluminescence assay (Immulite 2000, Siemens Healthcare Diagnostics Inc., UK).TRAb levels were determined only in GD and GO patients by electrochemiluminescence assay (Elecsys 2010, Roche Diagnostics, GER). Serum concentrations of alanine aminotransferase (ALT), aspartate aminotransferase (AST), total cholesterol (TC), high density lipoprotein (HDL) cholesterol, low density lipoprotein (LDL) cholesterol, creatinine, gamma GT, blood glucose and triglycerides were determined by enzymatic colorimetric methods (AU 5800, Beckman Coulter Inc., USA).

### Statistical analysis

Results are presented as mean and standard deviation or medians with quartile intervals when appropriate. A comparison of the data groups was performed using the following methods: Student T-test, Mann-Whitney and Kruskal-Wallis tests, and when necessary, a post Dunn test. Linear regression was performed for Se or SePP and TSH, FT4, FT3, TPOAb, TgAb and TRAb variables. Multiple regressions were performed considering Se or SePP as the dependent variable, and TSH, FT4, FT3, TPOAb, TgAb and TRAb as independent variables. Statistical significance was set at P < 0.05. All analyses were performed with Systat version 13 (Systat Software Inc., San Jose, CA. USA).

## RESULTS

**Figure 1:**
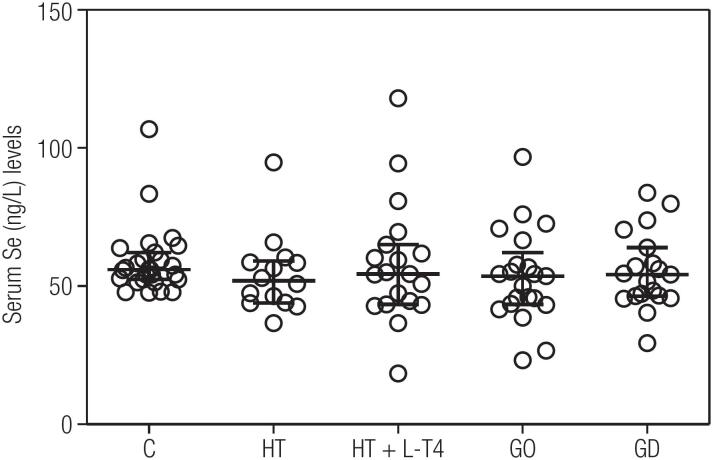
Individual serum selenium (Se) concentrations expressed as median and interquartile ranges. Control individuals (C), Hashimoto's thyroiditis (HT), Graves'disease (GD), Graves’ Ophthalmopathy (GO) and Hashimoto's thyroiditis levothyroxine (HT + LT4) patients. P value = 0.48; Kruskal-Wallis test.

**Figure 2:**
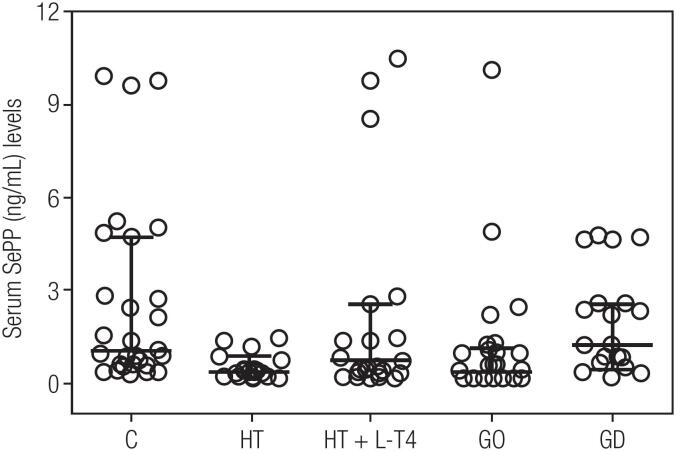
Individual serum selenoprotein P (SePP) concentrations expressed as median and interquartile ranges. Control individuals (C), Hashimoto's thyroiditis (HT), Graves’ disease (GD), Graves’ Ophthalmopathy (GO) and Hashimoto's thyroiditis levothyroxine (HT + LT4) patients.

**Figure 3:**
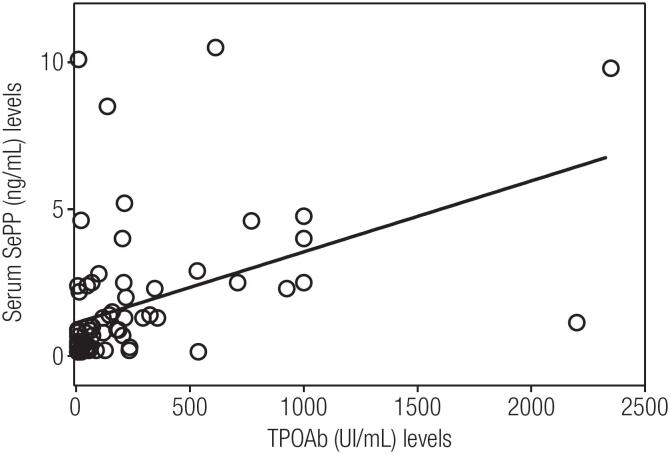
Linear regression analysis between Selenoprotein P (SePP) and thyroid peroxidase antibody (TPOAb): r = 0.445, r^2^ = 0.293, p < 0.0001, n = 73.

As shown in Table 2 the five groups were similar regarding age, gender, BMI, serum TSH, FT4 and TRAb. Serum FT3, TPOAb and TgAb levels was higher in GD patients (P < 0.0001). The serum biochemical characteristics of the five groups were similar regarding alanine aminotransferase, aspartate aminotransferase, creatinine, gamma-glutamyl transferase, glucose, low density lipoprotein cholesterol, high density lipoprotein cholesterol and triglycerides. Serum Se levels were similar among the studied groups (P = 0.48) as depicted in Figure 1, 56.0 (52.4-61.5 µg /L) in C; 54.2 (46.5-61.1 µg/L) in GD patients; 53.6 (43.5-60.0 µg/L) in GO patients, 51.9 (44.6-58.5 µg/L) in HT patients and 54.4 (44- 63.4 µg/L) in HT+LT4 patients. However, SePP serum was lower (P = 0.002) in GO patients; 0.30 (0.15-1.05 µg/mL) and in HT patients; 0.35 (0.2-1.17 µg/mL) compared to C; 1.00 (0.56-4.21 µg/mL), GD; 1.19 (0.622.5 µg/mL) and HT+LT4 0.7 (0.25-1.95) patients (Figure 2). Multiple regression analysis indicated no independent variables for either Se or SePP. However, there was a significant relationship between SePP and TPOAb as shown in Figure 3 (r coefficient = 0.445, R^2^ = 0.293; P < 0.0001).

**Table 2:** Clinical and laboratorial characteristics

	C (n = 27)	H T (n = 14)	G O (n = 21)	G D (n = 19)	HT+LT4 (n = 19)
Gender (women/men)	20/7	13/1	15/6	14/5	17/2
Age (yr.)*	51	46	58	54	52
	(39.2-66.2)	(30.2-64.5)	(44.7-66.5)	(48-58)	(46.5-55.5)
BMI (kg/m^2^)**	25.8 ± 3.8	26.6 ± 4.1	28.2 ± 4.49	27.1 ± 5.4	28.3 ± 5.3
TSH (mU/L)**	1.63 ± 0.72	3.00 ± 2.06	2.40 ± 2.09	1.45 ± 1.21	2.73 ± 2.39
FreeT4 (ng/dL)**	1.16 ± 0.20	1.23 ± 0.22	1.252 ± 0.279	1.278 ± 0.278	1.322 ± 0.237
FreeT3 (µg/mL)*	4.60	3.1	2.60^1^	3.15^1^	2.8^1^
	(3.65-5.20)	(2.8-3.45)	(2.55-3.0)	(2.57-3.37)	(2.6-3.05)
TgAb (UI/mL)*	11.9	71.9^2^	1.0^2^	1.0^2^	12.25^2^
	(10.0-19.5)	(1.00-170.0)	(1.0-1.0)	(1.0-12.0)	(1.0-87.9)
TPOAb (UI/mL)	5.0	79.45^3^	22.0^3^	58.5^3^	146^3^
	(5.0-9.70)	(47.32-209.7)	(7.0-62.75)	(16.5-405.5)^3^	(54.5-325)
TRAb (UI/L)			0.65	1.08	
			(0.30-4.86)	(0.41-3.92)	

* Median and interquartile intervals; 25% and 75%;

** Mean and standard deviation.

1 P < 0.0001 C vs. HT + LT4, C vs. GO, C vs. GD,

2 P < 0.0001 C vs. HT, C vs. HT + LT4, C vs. Go, C vs. GD,

3 P < 0.0001 HT vs. GO, HT vs. GD, HT + LT4 vs. GO, HT + LT4 vs. GD.

## DISCUSSION

In this study, the concentration of Se found in our subject sample was lower than in populations of some countries such as Japan, the United States, Turkey, England, Austria and Denmark, but was similar to values obtained in other countries such as Greece and China (2,12,20-26). Interestingly these countries are considered as Se deficient. Serum Se concentrations found in our normal subjects were similar to those obtained by Cardoso and cols. (27) in Sao Paulo, Brazil. However, our C group seems to have lower levels compared to previous studies conducted in Brazil which is reasonable for a marginally Se-deficient population. Environmental or dietary factors may explain these differences, taking into account the interval between the studies (28-31). Here, we observed lower Se levels in HT, GD, GO and HT+LT4 patients in comparison with the C group, but the differences were not statistically significant. The small numbers of studied patients may be the responsible. These data are in accordance with the results of Erdal and cols. (21), Moncayo and cols. (23) and Pedersen and cols. (12). A possible explanation may be the presence of patients with elevated serum TPOAb values (greater than 1,000 IU/mL) compared with our patients, which could lead to follicular thyroid cell damage, and consequently increased Se-dependent protein depletion resulting in low Se serum concentrations (31). The serum Se concentrations obtained in GD patients were similar to those reported by Zagrodzki and cols. (32), but lower than values obtained by Pedersen and cols. (12) in newly diagnosed (in the hyperthyroid phase) GD patients, which pointed out that inflammatory activity could be responsible for the low serum Se levels. Furthermore, all GD patients were euthyroid, in treatment with methimazole, which may have reduced inflammatory processes and cellular immunity, thereby further increasing Se levels. In contrast to our study, Khong and cols. (33) observed that Se serum values were lower in GD with GO than in GD without GO patients, and the most likely cause for this difference may be the inclusion of GO patients with mild to moderate GO in our study. The main finding of this study was significantly lower serum SePP in both HT and GD with GO patients, and in HT patients the levels were lower than that obtained by Eskes and cols. (34). One possible reason for this could be the use of different methodologies. The normal values of serum SePP in GD patients may be explained by the methimazole treatment that could have influenced the thyroid autoimmune system since the drug has an immunomodulatory action and decreases free radicals produced in thyroid follicular cells. The HT + LT4 group showed no significant difference in SePP serum, probably because thyroid function stabilized, which would decrease the consumption of selenium- dependent proteins. Thus, the finding of a low serum SePP values in HT and GO patients may be a result of inflammatory reactions associated with an increase in Se consumption in order to reduce the production of free radicals generated by an immunological attack. Limitations of our study consist in the low number of patients analyzed in the GD, HT and HT + LT4 groups, of the absence of GD patients without treatment in the hyperthyroid phase, the GO patient study group and the lack of serum GPx determinations. Further studies are needed to standardize benchmarks, as well as to improve methodologies used for serum Se and SePP concentration determinations. Leo and cols. recently demonstrated that Se supplementation does not have an adjuvant role in the short-term control of hyperthyroidism (35). However, further studies are also needed in order to demonstrate the probable changes in serum SePP in GD patients before and during treatment with or without Se supplementation. It should be noted that before Se supplementation it is important to establish the serum Se levels to provide improved effectiveness in GD and GO treatment. Thus, the determination of serum Se and SePP values could be used in assessing severity and inflammatory activity in AITD patients. In conclusion, our study found lower serum Se concentrations in the C group than those in other countries, but similar in other countries such as Greece and China countries considered as having marginally Se-deficient populations. Low serum SePP levels in both HT and GO patients may represent inflammatory reactions with a consequent increase in consumption of Se-dependent proteins in an attempt to prevent the production of free radicals generated by thyroid autoimmune aggression. In addition, serum SePP was related to thyroid immunity. This hypothesis may be further studied before indicating serum SePP as an effective biomarker of selenium status.
